# Assessment and prioritization of the WHO “best buys” and other recommended interventions for the prevention and control of non-communicable diseases in Iran

**DOI:** 10.1186/s12889-020-8446-x

**Published:** 2020-03-14

**Authors:** Ahad Bakhtiari, Amirhossein Takian, Reza Majdzadeh, Ali Akbar Haghdoost

**Affiliations:** 1 Department of Health Management and Economics, School of Public Health, Tehran University of Medical Sciences, Tehran, Iran; 2 Department of Global Health and Public Policy, School of Public Health, Tehran University of Medical Sciences, Tehran, Iran; 3 Health Equity Research Centre (HERC), Tehran University of Medical Sciences, Tehran, Iran; 4 Department of Epidemiology and Biostatistics, School of Public Health, Tehran University of Medical Sciences, Tehran, Iran; 5 Social Determinants of Health Research Center, Institute for Futures Studies in Health, Kerman University of Medical Sciences, Kerman, Iran

**Keywords:** Non-communicable diseases (NCDs), Preventive interventions, Multi-criteria decision analysis (MCDA), National action plan, Prioritization, Iran

## Abstract

**Background:**

The WHO’s “best buys” and other recommended interventions are a menu of policy options and cost-effective interventions for the prevention and control of major noncommunicable diseases (NCDs). The menu has six objectives, implementing which by member states is expected to promote the achievement of the nine NCD targets by 2025. In line with their context, countries can select from the menu of best buys and other recommended interventions. Iran adopted its national action plan on NCDs, 2015, including global as well as some specific goals and targets. This study had two objectives: analyzing the gaps to reach the national targets on NCDs; and prioritizing the best buys and other recommended interventions based on multi-criteria decision-making (MCDA) method for the context of Iran.

**Methods:**

This is a mixed-methods study. We used qualitative textual evidence (documentary content analysis) and MCDA for prioritization of interventions based on five criteria, including a number of people to be potentially affected by the intervention, cost-effectiveness of the intervention, attributable burden (DALY per 100,000), hospitalization and variations among income levels. Data related to five criteria for each intervention were extracted from national studies and relevant international organizations. The weight of each criterion determines based on the opinions of national experts.

**Results:**

Out of 105 actions and interventions recommended by WHO, only 12 of them were not on the national agenda in Iran, while the six missed interventions were related to objective number 4. Only one of the best buys Group’s interventions was not targeted (vaccination against human papillomavirus, two doses of 9–13-year-old girls), for which arrangements are being made for the implementation. Encouraging and educating healthy dietary habits and increasing public awareness about the side effects of smoking and exposure to second-hand smoke, e.g., through mass media campaigns, are among the interventions in need of serious prioritization. The priority of interventions was independently calculated in the area of risk factors and clinical preventive interventions.

**Conclusion:**

Due to limited resources, low and middle-income countries (LMICs) need to identify and prioritize more cost-effective and more equitable interventions to combat the NCD epidemic. Based on our findings, we advocate more investment in the mass and social media campaigns to promote a healthy diet, avoid tobacco use, as well as the inclusion of some effective clinical preventive interventions into the national action plan, along the long pathway to tackle NCDs and ultimately reach sustainable health development in Iran. The use of the MCDA approach assisted us in formulating a simultaneous use of efficiency and equity, and other indices for prioritizing the interventions.

## Background

**Fig. 1:**
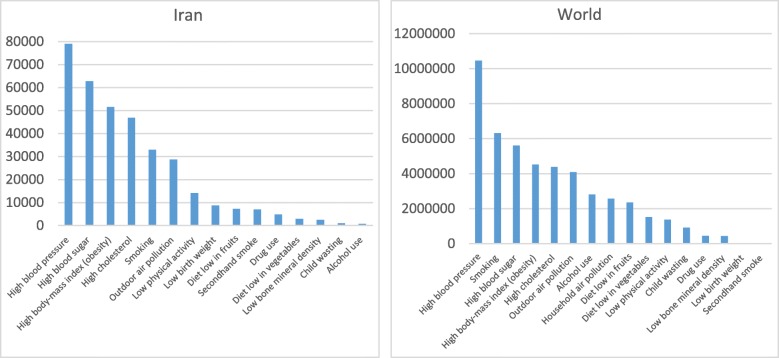
Number of deaths by risk factor, Iran/World, 2016, the total annual number of deaths by risk factor, measured across all age groups and both sexes [7]

The World Health Organization (WHO) has recommended a set of cost-effective and recommended interventions, in the format of six general objectives to combat non-communicable diseases (NCDs) [1]. So-called the WHO’s ‘best buys,’ they are considered as the gold standard and a strategic response to the worldwide tsunami of non-communicable diseases, through “saving lives, spending less” [2, 3] to reduce over 41 million annual deaths due to NCDs, including 15 million “premature” death (30–69 years old), over 85% of which occurring in low and middle-income countries (LMICs) [4–6]. Figure 1 presents the leading causes of death during recent decades in Iran and worldwide. Cumulative economic losses to LMICs resulted from four main NCDs are estimated to surpass US$ 7 trillion between 2011 and 2025 (an average of nearly US$ 500 billion per year), 70% of which will occur in upper-middle-income countries. It has been estimated that 51% of this annual loss will be related to cardiovascular diseases, which is equivalent to approximately 4% of these countries’ current annual output [8].

The four main groups of NCDs make the most substantial contribution to mortality among the Iranian population (Table 1). In the Eastern Mediterranean Region (EMR), it is estimated that more than 2.2 million people died from NCDs in 2008, representing 53% of total deaths. Furthermore, an estimated 35% (768000) of people who died from NCDs in this region were below 60 years, while morbidity due to NCDs accounts for over 60% of the regional disease burden [10].

**Table 1:** The status of four main NCDs, World and Iran, 2016 [9]

	The share of NCDs from total deaths. Both sexes. All age (%)		The share of NCDs from total DALYs. Both sexes. All age (%)		Mortality (% of total deaths), all ages, both sexes, 2016						
	1990	2017	1990	2017	Cardiovascular diseases	Cancers	Chronic respiratory diseases	Diabetes	Other NCDs	Communicable, maternal, perinatal, etc.	Injuries
World	57.72	73.41	43.19	62.06	31	16	7	3	15	20	9
Iran	50.11	82.32	45.33	76.6	43	16	4	4	15	8	10

In line with the Health Transformation Plan (HTP) began in 2014 in Iran, in 2015, the Ministry of Health and Medical Education (MoHME) established the Iranian Non-Communicable Diseases Committee (INCDC) [11]. Led by the minster, the INCDC aims to make evidence-based policies and monitor their appropriate implementation to reduce the mortality rate of NCDs by 30% until 2030. The INCDC prepared the National Action Plan for prevention and control of NCDs, which in 2016 was approved by the Supreme Council of Health and Food Security (SCHFS), led by the president. It also adapted WHO’s PEN (package of essential NCDs’ interventions for primary health care (PHC) in low-resource settings), so-called IraPEN 2015–25 strategy to enhance early detection of NCDs risk factors as well as mental health services within PHC network in Iran [12]. As a result of its performance, in 2016, the WHO identified Iran as a fast-track country. In 2018, the UN Interagency Task Force on NCDs (UNITAF) awarded INCDC [13] for its leading role in beating NCDs in Iran [14].

The WHO’s 2018 country profiles show that despite many interventions available for prevention and control of NCDs, more than 50% of countries will be likely to miss the 2030 NCD targets [9, 15], mainly due to limited resources. Despite some contradictory evidence about the status of four main NCDs in Iran [16], among 186 countries studied, Iran has been identified as moving in the right direction along the pathway towards achieving the 2030 NCD targets [15]. Therefore, it is crucial that policymakers become equipped with tools and skills to be able to select and prioritize appropriate interventions to combat NCDs [17]. WHO has identified a set of evidence-based “best buy” interventions that are not only highly cost-effective [18], but also are feasible and appropriate to be implemented within the constrained LMIC local health systems [8]. Cumulative cost and benefits of scaling up “Best Buy” interventions for cardiovascular diseases in 2011–2025 are about 120 US$ billions cost and 377 US$ billions of economic benefit [8], representing the cost-effectiveness of the interventions.

This study had two goals: 1) Identify gaps between WHO’s best buys and other recommended interventions with Iran’s national action plan and other policies on NCDs, to get them on the agenda; and 2) contextual prioritization of best buys and other recommended interventions based on MCDA in Iran. These two goals were combined to determine the position of non-targeted interventions during the process of final prioritization. While Iran has been gearing up its efforts to reach sustainable development goals (SDGs), our findings will help, we envisage, pave the way to achieve the goals of national action plans for prevention and control of NCDs, particularly SDG 3.4 to reduce 30% premature death due to NCDs by 2030 in the country.

## Methods

This is a mixed-method study. We used a comparative framework [19–21], to conduct document analysis for comparing the existing policies about NCDs in Iran with the WHO’s recommended interventions and policies on NCDs. MCDA quantitative approach was also used to do priority setting of preventive intervention in two areas: reducing modifiable risk factors and strengthening and reorientation of the health systems to address the prevention and control of NCDs. The following steps were followed to achieve these goals.

### Familiarization and identifying a framework for documentary content analysis

Our team scrutinized the ‘best buys’ and other recommended interventions for the prevention and control of NCDs [22]. The menu of policy options and cost-effective interventions has six objectives, 88 interventions, and 17 overarching/enabling actions (an updated version of 2017), categorized based on the risk factors, disease, cost-effectiveness, and type of intervention. We read the related documents on the WHO website several times for more familiarization. We also obtained and reviewed national documents of selected WHO’s fast track countries (i.e., Bhutan, Sri-Lanka, Philippines), with regards to the prevention and control of NCDs. Also, the senior author (AT) is a member of INCDC and had frequent and ongoing interactions with WHO, i.e., global meetings on NCDs as well as informal contacts with staff and representatives of many countries, from local to global levels. To design a comparative framework [19–21], we considered objectives and interventions for each of these objectives as a basis for comparison and matching (Table 2). Finally, we collected national policy and documents (see Appendix A) related to the identified objectives, actions, and interventions, from the MoHME and scrutinized them in the same way.

**Table 2:** A comparative framework for the content analysis of national documents

Objective	Actions and interventions ^a^	
1. To raise the priority accorded to the prevention and control of NCDs in global, regional and national agendas and internationally agreed development goals, through strengthened international cooperation and advocacy (1.1 to 1.4)	4 policy options	
2. To strengthen national capacity, leadership, governance, multisectoral action, and partnerships to accelerate country response for the prevention and control of NCDs. (2.1 to 2.4)	4 policy options	
3. Reducing modifiable risk factors for NCDs and underlying social determinants through creation of health-promoting environments (3.1 to 3.49)	TOBACCO	3 overarching/enabling actions 9 best-buys and other recommended interventions
	HARMFUL USE OF ALCOHOL	3 overarching/enabling actions 11 best-buys and other recommended interventions
	UNHEALTHY DIET	2 overarching/enabling actions 13 best-buys and other recommended interventions
	PHYSICAL INACTIVITY	1 overarching/enabling actions 7 best-buys and other recommended interventions
4. Strengthen and orient health systems to address the prevention and control of NCDs and the underlying social determinants through people-centered PHC and UHC (4.1 to 4.38)	OVERARCHING/ENABLING ACTIONS	8 actions
	CARDIOVASCULAR DISEASE	10 interventions
	DIABETES	7 interventions
	CANCER	7 interventions
	CHRONIC RESPIRATORY	6 interventions
5. To promote and support national capacity for high-quality research and development for the prevention and control of NCDs (5.1 to 5.5)	5 policy options	
6. To monitor the trends and determinants of NCDs and evaluate progress in their prevention and control (6.1 to 6.5)	5 policy options	

^a^Click to see the title of the interventions

### Indexing, charting, and interpretation

We codified the national documents (see Appendix A) manually and organized them in the comparative framework. All documents were obtained from the MoHME and other ministries and were official and authentic; hence their credibility was ensured. Three scenarios were drawn accordingly:

1. A label: If an intervention (105 actions and interventions recommended by WHO numbering from1.1 to 6.5) especially reflected in Iran’s National action plan, it was labeled as green.
2. B label: If interventions were not mentioned in the National Action Plan but mentioned in other national documents or policies, they were labeled blue.
3. C label: Interventions that were not mentioned in any related documents or policies were labeled as red.

Finally, we requested the INCDC to confirm the findings to ensure the accuracy of the results (see Appendix B).

### Quantitative analysis

**Fig. 2:**
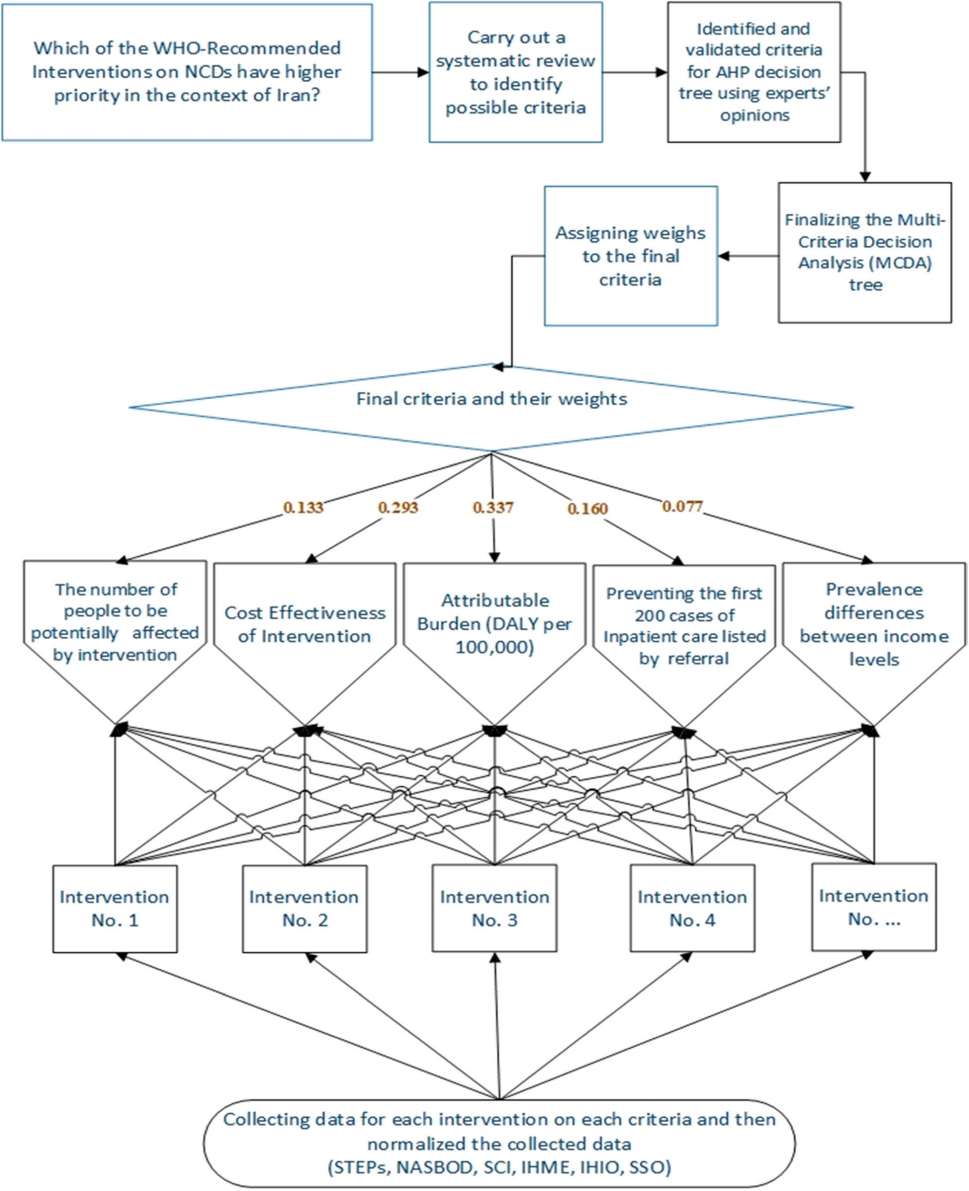
Development and application of a multi-criteria priority setting algorithm in Iran

We used MCDA to identify which WHO-Recommended Interventions on NCDs would have a higher priority in the context of Iran (Fig. 2).

Interventions belonging to objectives 1, 2, 5, and 6 were general non-quantifiable recommendations, and along with nine interventions of objectives 3 and 8 of objective 4, were excluded from the prioritization process. Finally, 40 interventions of objective 3 and 30 interventions of objective 4 were included in the prioritization process. We conducted an analysis of the prioritization process for objectives 3 and 4 independently. This was because the nature of interventions in objective 3 was based on the risk factors, while interventions in objective 4 were based on the clinical processes.

We carried out a systematic review (see Appendix C) and explored the opinions of experts to determine the list of possible criteria for MCDA. We reviewed the title and abstract of 1180 articles that were published between 2000 until 2018, 24 of which were entered into the final phase [23–46] towards the identification of potential criteria for the prioritization process.

The criteria used in the reviewed studies divide into thirteen classes, and according to experts, these cases were increased to 14 cases. Finally, five of them were selected for this study by the experts and study teams. We then measured the validity and weighted the listed criteria to prioritize interventions through consultation with a group of eight national experts, whom we selected purposefully, All of them are faculty members, and six of them had ten to twenty-five years of research experience (Table 3), followed by finalizing the MCDA tree (Table 4). Pairwise comparisons were used to weigh the selected criteria by a team of experts.

**Table 3:** Knowledge and experience of selected experts

Experts	Knowledge / Expertise / Experience / Position	Level
1	Global Health & Policy, Health Equity, Public Policy, NCDs	International
2	Health Equity, Epidemiology, and Biostatistics, Top Level Leadership and Adviser of The MoHME,	National
3	Health Economics, Public Health	National
4	Director of NCDs National Research Center, Burden of Diseases	National
5	NCDs National Research Center	National
6	Director of The Ministry of Health’s Center for NCDs	National
7	Social Determinants of Health, Public Health	Provincial
8	Health Policy, Health Equity, Health Economics	Provincial

**Table 4:** The MCDA tree final criteria and their weight

	Criteria	Weight	Data on each intervention
1	Number of people to be potentially affected by intervention	.133	3.1 to 3.49 and 4.1 to 4.38 (excluded interventions: 3.1–3.2-3.3-3.13-3.14-3.15-3.27-3.28-3.42- and 4.1 to 4.8)
2	Cost-effectiveness of intervention	.293	
3	Attributable burden (Daly per 100,000)	.337	
4	The 200 disease codes that led to the largest hospitalization in the whole country over a year. The chance that the intervention may prevent some of them based on the model of 4 diseases, 4 modifiable shared risk factors	.160	
5	Prevalence differences between income levels	.077	
	Inconsistency = 0.01 with 0 missing judgments		

The inconsistency measure is valuable for identifying likely errors in judgments as well as actual inconsistencies in the judgments themselves. In general, the inconsistency ratio should be less than 0.1 to be considered reasonably consistent [47]. In this study, the inconsistency rate for the comparison matrix of criterion relative to the objective was equal to 0.01.

### Data collection for each intervention in the five selected criteria

In this step, we collected data regarding the five selected criteria for each intervention. Those related to the first and Fifth criteria were obtained from three sources: national survey of NCDs’ risk factors in 2016 (STEPs) [48]; NASBOD (National and Sub-National Burden Of Diseases, injuries, and risk factors) in 2015 [49, 50]; and the Statistical Centre of Iran (SCI) [51]. The cost-effectiveness of interventions has been set by WHO; When assessing the cost-effectiveness of interventions by WHO, twenty countries were selected, one of which was Iran [1]. We obtained the burden of disease and risk factor data from the Institute for Health Metrics and Evaluation (IHME) [52, 53]. Finally, for criterion four, the WHO model “4 diseases, 4 modifiable shared risk-factors” [54] (Table 5) was used to determine which interventions prevent inpatient care related to four main NCD.

**Table 5:** The 4 diseases, 4 modifiable shared risk-factors

	Tobacco use	Unhealthy diets	Physical inactivity	Harmful use of alcohol
**Cardio-vascular**	**√**	**√**	**√**	**√**
**Diabetes**	**√**	**√**	**√**	**√**
**Cancer**	**√**	**√**	**√**	**√**
**Chronic respiratory**	**√**			

We obtained the inpatient care data, based on the diagnostic ICD-10 code, related to four main NCD disease groups from two main public insurance organizations in Iran (Iranian Health Insurance Organization (IHIO) and Social Security Organization (SSO)), which together cover over 90% of the country’s population. More than 2.4 million inpatient care (over 10,000 diagnostic codes in 2017) were arranged based on the number of admissions. Regarding the WHO model, the sum of the referrals according to diagnostic codes related to four main NCDs diseases (Table 6) (cardiovascular, diabetes, cancer, and chronic respiratory diseases) were considered for relevant preventive interventions. Data in each column for criteria were normalized (each intervention value was divided by the sum of the whole column of a criterion) and entered to the expert choice software version 11 in a distributive Mode.

**Table 6:** Inpatient care data related to four main NCD diseases

Disease group	ICD 10 Code ^a^	Number of inpatient admissions	sum	percentage of total admissions
Cardiovascular diseases	I20.0 I25.1 I10 R07.4 I64 I50.0 I25.9 I21.9 I48 I50.9 I80.2 I51.6 G45.9 Z03.5 I24.9	56,238 29,660 26,819 16,363 12,663 11,763 11,437 10,174 7187 6906 4048 3794 3412 3012 2347	205,823	11.99
Cancers	Z51.1 N83.2 N63 D48.7	21,301 5645 2242 710	29,898	1.74
Diabetes	J35.3 O24.4 E11.5 E14.5 E10.9 E11.9	8531 5139 4013 1994 1933 1190	22,800	1.32
Chronic respiratory diseases	J18.9 J44.9 R06.0 J45.9 J44.1 J21.9 J40 J46	44,902 16,173 12,856 12,367 3281 2486 2021 966	95,052	5.54

^a^33 code out of the first 200 code related to 4 NCDs

## Results

More than 65% of the missed interventions were related to objective 4. From 105 actions and interventions recommended by WHO, only 12 cases were not targeted in Iran (Table 7); with four of which being overarching/enabling policy interventions, four being other recommended interventions by WHO (cost-effective analysis not available), three being Effective interventions with cost-effectiveness analysis >I$ 100 per DALY averted in LMICs, and one being “best buys”: effective interventions with cost-effectiveness analysis ≤ I$ 100 per DALY averted in LMICs.

**Table 7:** Missed interventions for prevention and control of NCDs in Iran

Objective	Missed interventions	Type of intervention
1	1.3 Strengthen international cooperation for resource mobilization, capacity-building, health workforce training and exchange of information on lessons learned and best practices	Overarching/enabling policy interventions.
2	2.2 Assess national capacity for prevention and control of NCDs	
4	4.2 Explore viable health financing mechanisms and innovative economic tools supported by evidence	
4	4.7 Develop and implement a palliative care policy, including access to opioids analgesics for pain relief, together with training for health workers	
3	3.38 Limiting portion and package size to reduce energy intake and the risk of overweight/obesity	Other recommended interventions from WHO guidance (cost-effective analysis not available).
3	3.45 Ensure that macro-level urban design incorporates the core elements of residential density, connected street networks that include sidewalks, easy access to a diversity of destinations and access to public transport	
4	4.16 Anticoagulation for medium-and high-risk non-valvular atrial fibrillation and for mitral stenosis with atrial fibrillation	
4	4.31 Oral cancer screening in high-risk groups (for example, tobacco users, betel-nut chewers) linked with timely treatment	
4	4.10 Treatment of new cases of acute myocardial infarction** with either: acetylsalicylic acid, or acetylsalicylic acid and clopidogrel, or thrombolysis, or primary percutaneous coronary interventions (PCI)	Effective interventions with cost-effectiveness analysis >I$ 100 per DALY averted in LMICs.
4	4.12 Primary prevention of rheumatic fever and rheumatic heart diseases by increasing appropriate treatment of streptococcal pharyngitis at the primary care level	
4	4.13 Secondary prevention of rheumatic fever and rheumatic heart disease by developing a register of patients who receive regular prophylactic penicillin	
4	4.26 Vaccination against human papillomavirus (2 doses) of 9–13-year-old girls	‘Best buys’: Effective interventions with cost-effectiveness analysis = I$ 100 per DALY averted in LMICs.

Among WHO-recommended interventions that have been addressed, 49 interventions were directly mentioned in Iran’s national action plan and others mentioned and tracked in related policy and documents. The status of one of the interventions was ambiguous. Intervention 3.9 (advisory, therapeutic, and pharmaceutical interventions for smoking cessation) has been mentioned in the national documents, without any debate as to how to cover the costs.

**Fig. 3:**
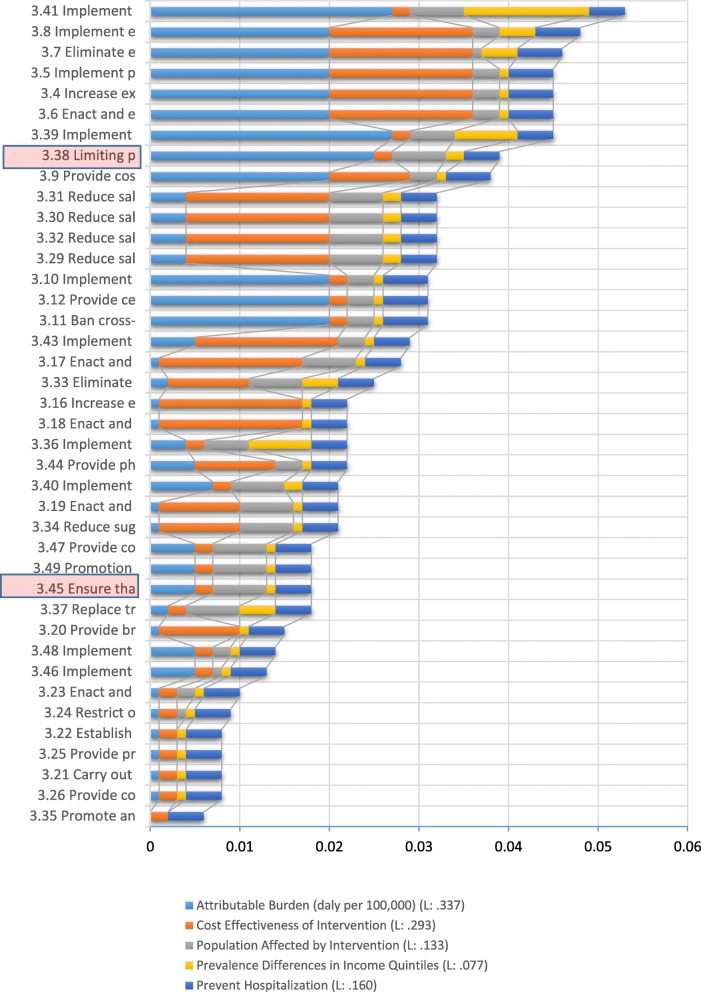
Prioritized interventions in the risk factors area (criteria 1: Attributable burden, weight: L: 0.337)

There were some missed interventions among total 49 interventions, which belonged to objective 3, i.e., interventions 3.38 and 3.45, both of which require intersectoral collaboration for food packaging and macro-level urban designing for implementation, respectively. Encouraging and educating healthy dietary habits and increasing public awareness about the side effects of smoking and exposure to second-hand smoke through mass media, are the essential interventions among 40 interventions of objective 3 to be prioritized in Iran. The third, fourth, fifth, sixth, and ninth priorities are related to tobacco use, especially exposure to secondhand smoke. The seventh, eighth, tenth, eleventh, twelfth, and thirteenth priorities are also related to controlling nutrition risk factors. Figure 3 demonstrates prioritized interventions. Untracked interventions in Iran are highlighted in red. See Table 8 for the full name of the The priority in each criterion for each intervention indicates in different colors Fig. 3. The final priority of each intervention was created based on the sum score of five criteria. (please see Table 8 for a full title of interventions).

**Table 8:** Full name of interventions belonged to objective three and Fig. 3

Code	Full name of the interventions that prioritized in Fig. 3
3.41	Implement mass media campaign on healthy diets, including social marketing to reduce the intake of total fat, saturated fats, sugars, and salt, and promote the intake of fruits and vegetables
3.8	Implement effective mass media campaigns that educate the public about the harms of smoking/tobacco use and second-hand smoke
3.7	Eliminate exposure to second-hand tobacco smoke in all indoor workplaces, public places, and public transport
3.4	Increase excise taxes and prices on tobacco products
3.5	Implement plain/standardized packaging and/or large graphic health warnings on all tobacco packages
3.6	Enact and enforce comprehensive bans on tobacco advertising, promotion, and sponsorship
3.39	Implement nutrition education and counseling in different settings (for example, in preschools, schools, workplaces, and hospitals) to increase the intake of fruits and vegetables
3.38	Limiting portion and package size to reduce energy intake and the risk of overweight/obesity
3.9	Provide cost-covered, effective and population-wide support (including brief advice, national toll-free quitline services) for tobacco cessation to all those who want to quit
3.29	Reduce salt intake through the reformulation of food products to contain less salt and the setting of target levels for the amount of salt in foods and meals
3.30	Reduce salt intake through the establishment of a supportive environment in public institutions such as hospitals, schools, workplaces, and nursing homes, to enable lower sodium options to be provided
3.31	Reduce salt intake through a behavior change communication and mass media campaign
3.32	Reduce salt intake through the implementation of front-of-pack labeling
3.10	Implement measures to minimize illicit trade in tobacco products
3.11	Ban cross-border advertising, including using modern means of communication
3.12	Provide cessation for tobacco cessation to all those who want to quit
3.43	Implement community-wide public education and awareness campaign for physical activity which includes a mass media campaign combined with other community-based education, motivational and environmental programs aimed at supporting behavioral change of physical activity levels
3.17	Enact and enforce bans or comprehensive restrictions on exposure to alcohol advertising (across multiple types of media)
3.33	Eliminate industrial trans-fats through the development of legislation to ban their use in the food chain
3.44	Provide physical activity counseling and referral as part of routine primary health care services through the use of a brief intervention
3.16	Increase excise taxes on alcoholic beverages
3.18	Enact and enforce restrictions on the physical availability of retailed alcohol (via reduced hours of sale)
3.36	Implement subsidies to increase the intake of fruits and vegetables
3.19	Enact and enforce drink-driving laws and blood alcohol concentration limits via sobriety checkpoints
3.34	Reduce sugar consumption through effective taxation on sugar-sweetened beverages
3.40	Implement nutrition labeling to reduce total energy intake (kcal), sugars, sodium and fats
3.37	Replace trans-fats and saturated fats with unsaturated fats through reformulation, labeling, fiscal policies or agricultural policies
3.45	Ensure that macro-level urban design incorporates the core elements of residential density, connected street networks that include sidewalks, easy access to a diversity of destinations and access to public transport
3.47	Provide convenient and safe access to quality public open space and adequate infrastructure to support walking and cycling
3.49	Promotion of physical activity through organized sports groups and clubs, programs and events
3.20	Provide brief psychosocial intervention for persons with hazardous and harmful alcohol use
3.46	Implement a whole-of-school program that includes quality physical education, availability of adequate facilities and programs to support physical activity for all children
3.48	Implement multi-component workplace physical activity programs
3.23	Enact and enforce an appropriate minimum age for purchase or consumption of alcoholic beverages and reduce the density of retail outlets
3.24	Restrict or ban promotions of alcoholic beverages in connection with sponsorships and activities targeting young people
3.21	Carry out regular reviews of prices in relation to the level of inflation and income
3.22	Establish minimum prices for alcohol where applicable
3.26	Provide consumer information about, and label, alcoholic beverages to indicate, the harm related to alcohol
3.25	Provide prevention, treatment, and care for alcohol use disorders and comorbid conditions in health and social services
3.35	Promote and support exclusive breastfeeding for the first 6 months of life, including the promotion of breastfeeding

**Fig. 4:**
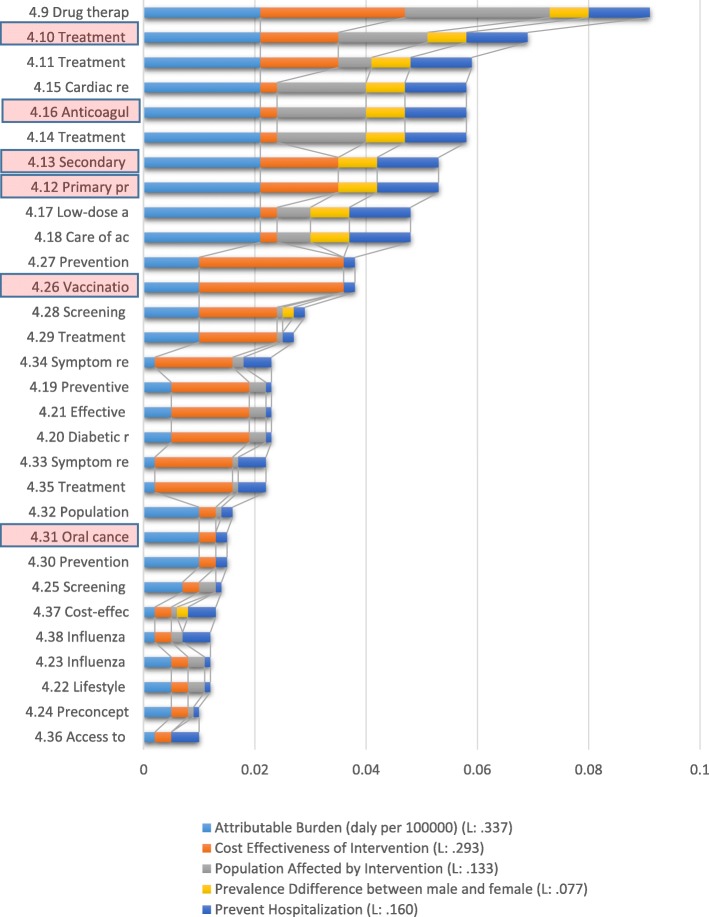
Prioritized recommended interventions for main NCDs

Six out of the total 38 interventions related to objective 4 were missed, and five of the missed interventions were among the first 12 prioritized interventions. Second-level prevention (intervention number 4.9) was used in Iran’s NCDs policies, while the third-level prevention after new cases of acute myocardial infarction (intervention number 4.10) has not been targeted overall. Primary (4.12) and secondary (4.13) prevention of rheumatic fever and rheumatic heart disease, although ignored, are of high priority. Treatment of acute ischemic stroke with intravenous thrombolytic therapy (4.11) and cardiac rehabilitation post-myocardial infarction are the interventions with high priority, which are appropriately met in the relevant policies in Iran (Fig. 4). See Table 9 for the full name of the interventions.

**Table 9:** Full name of interventions belonged to objective four and Fig. 4

Code	Full name of the interventions that prioritized in Fig. 4
4.9	Drug therapy (including glycaemic control for diabetes mellitus and control of hypertension using a total risk* approach) and counseling to individuals who have had a heart attack or stroke and to persons with high risk (≥ 30%) of a fatal and non-fatal cardiovascular event in the next 10 years
4.10	Treatment of new cases of acute myocardial infarction** with either: acetylsalicylic acid, or acetylsalicylic acid and clopidogrel, or thrombolysis, or primary percutaneous coronary interventions (PCI)
4.11	Treatment of acute ischemic stroke with intravenous thrombolytic therapy
4.15	Cardiac rehabilitation post-myocardial infarction
4.16	Anticoagulation for medium-and high-risk non-valvular atrial fibrillation and for mitral stenosis with atrial fibrillation
4.14	Treatment of congestive cardiac failure with angiotensin-converting-enzyme inhibitor, beta-blocker, and diuretic
4.13	Secondary prevention of rheumatic fever and rheumatic heart disease by developing a register of patients who receive regular prophylactic penicillin
4.12	Primary prevention of rheumatic fever and rheumatic heart diseases by increasing appropriate treatment of streptococcal pharyngitis at the primary care level
4.17	Low-dose acetylsalicylic acid for ischemic stroke
4.18	Care of acute stroke and rehabilitation in stroke units
4.27	Prevention of cervical cancer by screening women aged 30–49 years
4.26	Vaccination against human papillomavirus (2 doses) of 9–13-year-old girls
4.28	Screening with mammography (once every 2 years for women aged 50–69 years) linked with timely diagnosis and treatment of breast cancer
4.29	Treatment of colorectal cancer stages I and II with surgery +/− chemotherapy and radiotherapy
4.34	Symptom relief for patients with chronic obstructive pulmonary disease with inhaled salbutamol
4.19	Preventive foot care for people with diabetes (including educational programs, access to appropriate footwear, multidisciplinary clinics
4.21	Effective glycaemic control for people with diabetes, along with standard home glucose monitoring for people treated with insulin to reduce diabetes complications
4.20	Diabetic retinopathy screening for all diabetes patients and laser photocoagulation for prevention of blindness
4.33	Symptom relief for patients with asthma with inhaled salbutamol
4.35	Treatment of asthma using low dose inhaled beclometasone and short-acting beta-agonist
4.32	Population-based colorectal cancer screening, including through a faecal occult blood test, as appropriate, at age > 50 years, linked with timely treatment
4.31	Oral cancer screening in high-risk groups (for example, tobacco users, betel-nutchewers) linked with timely treatment
4.30	Prevention of liver cancer through hepatitis B immunization
4.25	Screening of people with diabetes for proteinuria and treatment with angiotensin-converting enzyme inhibitor for the prevention and delay of renal disease
4.37	Cost-effective interventions to prevent occupational lung diseases, for example, from exposure to silica, asbestos
4.38	Influenza vaccination for patients with chronic obstructive pulmonary disease
4.23	Influenza vaccination for patients with diabetes
4.22	Lifestyle interventions for preventing type 2 diabetes
4.24	Preconception care among women of reproductive age who have diabetes including patient education and intensive glucose management
4.36	Access to improved stoves and cleaner fuels to reduce indoor air pollution

## Discussion

The global action plan for Prevention and Control of NCDs has six objectives (Table 2), implementing which will facilitate the achievement of the nine NCD targets in member states by 2030, in line with the pathway towards SDG 3, SDG 3.4 in particular. Countries can select from the list of best buys and other recommended interventions, based on their national context [22].

Our study revealed that most WHO recommended interventions were considered in Iran’s national action plan. In particular, began in July 2015, the third phase of HTP included the following stages for smooth implementation of WHO’s best buys:

- In line with the WHO global action plan, the SCHFS, led by President, approved the National Action plan for prevention and control of NCDs [55].
- The INCDC [11], led by the minister of health, was established within the MoHME. The INCDC is the highest decision- making body in the health system to plan, monitor, and lead the country toward a 30% reduction in NCDs-related mortality by 2030.
- The INCDC prepared a number of national standard frameworks to reduce NCDs’ risk factors through multisectoral collaboration. It also approved the IraPEN 2015–25 strategy, which includes several cost-effective interventions for early detection of three common cancers (colorectal, breast, and cervix), accompanied by active risk score assessment for cardiovascular diseases and their appropriate management [55] within the PHC network.

Focusing on single criteria decision analysis for prioritizing feasible best buys and other relevant interventions could be misleading. MCDA can help create a rational priority setting process to inform national policymakers of reliable ways to take actions that can lead to meaningful outcomes in NCD’s prevention and control, which are tailored to their settings.

The highest priority intervention in our study is related to unhealthy diet, intervention number 3.41: *“Implement mass media campaign on healthy diets, including social marketing to reduce the intake of total fat, saturated fats, sugars, and salt; and to promote the intake of fruits and vegetables.”* Apart from tobacco-related interventions, most prioritized interventions are related to nutrition.

A recent study found that poor diet was responsible for more than 1 in 5 deaths globally, making the diet more deadly than tobacco and high blood pressure, with almost 11 million deaths per year due to an unhealthy diet [56]. Results of a systematic review presented three dietary patterns among Iranian society: healthy pattern, western pattern, and traditional pattern. Adherence to the healthy dietary pattern is expected to have a protective effect on NCDs. The Western dietary pattern was highly associated with NCDs [57]. HTP initiated some interventions to reduce the unhealthy diet in Iran, some of which discussed below.

In terms of regulations, the national standards for salt, sugar, and fat in processed food were revised as follows: salt intake to be reduced from 2.3 to 1%, while sugar and carbonate in industrial juices to be reduced to less than 10%. The SCHFS approved a reduction of palm oil import by 30% as well as the use of trans fatty acids in the confectionery and chocolate industry to less than 10% [58–61]. More than 1400 nutrition counselors were hired within PHC centers. Besides, national media campaigns were initiated to modify the pattern of salt, sugar, and fat consumption. A number of bilateral agreements were signed between the MoHME and other governmental departments and ministries to foster required multisectoral collaboration to address social and commercial determinants of NCDs [62].

Preventive tobacco-related interventions have a top priority in Iran, as also endorsed in related national policies. Nonetheless, when it comes to implementation, despite the MoHME’s recent efforts to increase imported and retail tobacco taxes, such taxes in Iran have consistently been among the lowest globally [63]. Let alone, illicit trade in tobacco exacerbates the burden of tobacco use in Iran and the region [64]. The good news is that over 55,000 schools have joined the tobacco awareness campaign, and many animations have been produced in this regard [65].

Selling alcoholic beverages is a crime in Iran, while advertising-related interventions are illegal (3.24). As a result, the legal age for the purchase of alcohol, drink-driving laws and physical access to alcoholic beverages are being implemented, and also counseling and treatment services are considered for harmful use of alcohol, a considerable proportion of which is either smuggled into the country or is non-standardized handmade [66], consuming which may lead to serious complications [67]. Nevertheless, with the expansion of the globalization of Muslim majority countries, there would be more alcohol-related challenges to be addressed [68].

In objective 4, interventions for combating cardiovascular disease are of the highest priority. In addition, four of these interventions (4.10, 4.16, 4.13, and 4.12) with top priority have not been mentioned in the national policies. A comprehensive strategy is critical to address cardiovascular disease, the strategy that combines preventive interventions into multifariousness factors, including behavioral, biological, psychosocial, health systems, and intersectoral factors. The combination of factors should be adjusted with regard to the country or the regional context [55]. The intervention 4.26 *“vaccination against human papillomavirus (2 doses) of 9–13-year-old girls”* is being pursued by the MoHME, while its adoption and implementation are yet to be accomplished.

MCDA has been used in the past to prioritize interventions in a variety of areas, e.g., HIV/AIDS [69], health interventions in the universal health coverage benefit package [70], interventions for chronic non-cancer pain [71], obesity research and prevention [72], respiratory, mental, children’s health, cardiovascular, and cancer interventions [40]. Combining different stakeholders’ views and balancing the benchmark between efficiency and equity in decision making and policymaking are among the benefits of using MCDA for prioritizing health-care decisions [73], as we also saw in this study.

### Study limitations

This study had two primary limits. First, valid data sources for criteria such as a continuation of the intervention effect, side effects, and acceptability were lacking; hence we did not enter them into the decision tree. Second, some interventions based on the objectives of Appendix 3 of the WHO Global NCD action plan were not quantifiable based on the selected criteria. WHO has identified these points as overarching/enabling actions. See the link at the bottom of Table 2.

### Policy recommendations

- Restrictions on resources in all countries, especially in the LMICs, require that programs be directed towards priority interventions. MCDA is a useful tool to help national policymakers for prioritizing the interventions. For example, this study found that nutrition-centered interventions had a higher priority compared to other interventions.
- The MCDA can also help local policymakers to tailor appropriate interventions into NCDs’ national programs, based on their contextual characteristics.
- Stakeholders’ conflict of interest might slow down the progress of healthcare interventions. Through including different and even contradicting philosophical views of decision-makers, joint weighing methods used in MCDA can balance and weight criteria for prioritization.
- We encourage insurance organizations to utilize group risk assessment and MCDA models to identify priority interventions for different groups of the population.

## Conclusion

This study has documented the utility of MCDA and framework approach for prioritizing NCDs interventions in Iran. Our analysis revealed a combination of different criteria with various philosophical perspectives in prioritizing and deciding about NCDs and priorities. An analysis of the framework in this study revealed missing interventions in the national action plan. The information system is considered the MCDA’s power supply system. The more metrics and detailed information provided, the more reasonable decisions may be made.

The 72nd world health assembly, held on May 21st 2019, reaffirmed the importance of preventing NCDs and called on world health leaders to consider them as an important pillar to achieving UHC [74]. Detailed information about direct and indirect costs of prevention and control of NCDs, the number of inpatient and outpatient visits, the total lost productivity and the economic burden of disease, are crucial dimensions for countries to make sensible decisions and encourage policymakers towards more investment in preventive interventions. Despite its limits, the MCDA model is helpful for prioritizing NCDs’ related intervention and take more effective steps to beat NCDs. NCDs are complex, multi-dimensional, and expensive to tackle. Unless the health system leaders can prioritize appropriate preventive interventions, the NCDs’ battle is hard to defeat, so UHC and sustainable health development might be difficult to achieve.

## Data Availability

The datasets used and/or analyzed during the current study are available from the corresponding author upon reasonable request.

## Appendix A

**Table:**

National policy and documents	IraPEN, National Action Plan for Prevention and Control of Non-Communicable Diseases and the Related Risk Factors in the Islamic Republic of Iran, 2015–2025, National Physical Activity Plan for Health Promotion in IR Iran, National Diabetes Prevention and Control Program, Comprehensive Tobacco Control Act, National Nutrition and Food Security Document 2012–2020, Comprehensive program for prevention, treatment and reduction of alcohol-related toxicity, National policies related to sugar, salt, and fat, National guidelines for COPD, prevention, diagnosis and treatment guidelines, National policies related to physical activity, nutrition, tobacco, alcohol, and clinical services

## Appendix B

**Table:**

C label: not mentioned in any related documents	1.3 & 2.2 & 4.2 & 4.7 & 3.38 & 3.45 & 4.16 & 4.31 & 4.10 & 4.12 & 4.13 & 4.26
A and B Labels: mentioned in the National Action Plan or in other national documents or policies	All WHO recommended interventions in Appendix 3 except C label

## Appendix C

**Table:**

PubMed: ((multiple criteria decision analysis [Title/Abstract] OR multiple-criteria decision-making [Title/Abstract]) OR TOPSIS [Title/Abstract]) OR analytic hierarchy process [Title/Abstract] AND (““loattrfull text””(24) AND (““2000/01/01””[PDA: ““2018/12/31””[PDAT]) AND ““humans””[MeSH Terms] AND English [lang]) Embase: Embase, MEDLINE Query(‘multicriteria decision analysis’/de OR ‘mcda (multiple criteria decision making)’:ti,ab OR ‘mcdm (multiple criteria decision making)’:ti,ab OR ‘multi criteria decision analysis’:ti,ab OR ‘multi criteria decision making’:ti,ab OR ‘multicriteria decision analysis’:ti,ab OR ‘multicriteria decision making’:ti,ab OR ‘multiple criteria decision analysis’:ti,ab OR ‘multiple criteria decision making’:ti,ab OR topsis:ti,ab OR ‘analytic hierarchy process’/exp) AND (2000:py OR 2001:py OR 2002:py OR 2003:py OR 2004:py OR 2005:py OR 2006:py OR 2007:py OR 2008:py OR 2009:py OR 2010:py OR 2011:py OR 2012:py OR 2013:py OR 2014:py OR 2015:py OR 2016:py OR 2017:py OR 2018:py) AND ‘article’/it 979 Embase + 284 PubMed = 1263–83 duplicate = 1180 articles - Exclusion rules (use of MCDA outside of the health area; and article does not mention the criteria used in MCDA.) = 24 articles Criteria identified for possible use in MCDA: Efficiency and effectiveness, Reveal the results of intervention, Continuity of effect, Side effects and acceptability, Credibility or quality of documentation, Degree of need, The number of times the process needs to be repeated, Cost of intervention, Cost-effectiveness, Advantage for the health system, Burden of Disease, Number of population affected and Inpatient admissions, Alternatives, and Inequality.	

## Abbreviations

DALY: Disability-Adjusted Life Year
EMR: Eastern Mediterranean Region
HTTP: Health Transformation Plan
IHIO: Iranian Health Insurance Organization
IHME: Institute for Health Metrics and Evaluation
INCDC: Iranian Non-Communicable Diseases Committee
LMICs: Low and Middle Income Countries
MCDA: Multi-Criteria Decision Analysis
MoHME: Ministry of Health and Medical Education
NASBOD: National and Sub-National Burden Of Diseases
NCDs: Noncommunicable Diseases
PHC: Primary Health Care
SCHFS: Supreme Council of Health and Food Security
SCI: Statistical Centre of Iran
SDGs: Sustainable Development Goals
SSO: Social Security Organization
UHC: Universal Health Coverage
UNITAF: UN Interagency Task Force
WHO: World Health Organization
